# Pulmonary tuberculosis: Tomographic evaluation in the active and post-treatment phases

**DOI:** 10.1590/S1516-31802003000500004

**Published:** 2003-09-01

**Authors:** Sidney Bombarda, Cláudia Maria Figueiredo, Márcia Seiscento, Mário Terra

**Keywords:** Tuberculosis, Computed x-ray tomography, Diagnosis, Tuberculose, Tomografia computadorizada por raios x, Diagnóstico

## Abstract

**CONTEXT:**

Adequate knowledge of images consistent with tuberculosis activity is an important resource for tuberculosis diagnosis and treatment.

**OBJECTIVE:**

To evaluate the structural alterations caused by tuberculosis in the pulmonary parenchyma, both during the active phase of the disease and after the end of the treatment, through computerized tomography of the thorax.

**TYPE OF STUDY:**

Prospective study.

**SETTING:**

Pulmonary Division, Hospital das Clínicas, Faculdade de Medicina da Universidade de São Paulo.

**PARTICIPANTS:**

20 patients, carriers of pulmonary tuberculosis, confirmed by Mycobacterium tuberculosis culture.

**PROCEDURES:**

Conventional tomography scans of the patients were obtained at two times: upon diagnosis and after the end of the treatment. The following were considered suggestive signs of tuberculosis activity: centrilobular nodules with segmented distribution, confluent micronodules, consolidations, thick-walled cavities, nodules, masses, thickening of the bronchial walls, tree-in-bud appearance and cylindrical bronchiectasis.

**MAIN MEASUREMENTS:**

The presence of suggestive signs of tuberculosis activity was compared between the start and the end of treatment by means of the signs test (z).

**RESULTS:**

All patients (20/20) presented suggestive signs of tuberculosis activity at the start of treatment. After the end of treatment, 13 patients (13/20) still presented some suggestive signs consistent with activity. A reduction in the extent of lung attack was seen post-treatment, in relation to its start (z = 10.10). This change was statistically significant (p < 0.001).

**CONCLUSION:**

Signs suggestive of tuberculosis activity are present in the active disease and are seen via computed tomography. The extent of parenchymal attack significantly decreases following treatment. Such signs may be useful in the diagnosis of pulmonary tuberculosis.

## RESUMO

**CONTEXTO:**

O adequado conhecimento das imagens compatíveis com atividade da tuber-culose é um importante recurso para o seu diagnóstico e acompanhamento.

**OBJETIVO:**

Avaliar as alterações estruturais da tuberculose no parênquima pulmonar, durante a fase ativa da doença e após o término do tratamento, através da tomografia computadorizada do tórax.

**TIPO DE ESTUDO:**

Estudo prospectivo.

**LOCAL:**

Disciplina de Pneumologia da Faculdade de Medicina da Universidade de São Paulo.

**PARTICIPANTES:**

20 pacientes portadores de tuberculose pulmonar confirmadas por cultura de *Mycobacterium tuberculosis.*

**PROCEDIMENTOS:**

Foram obtidas tomografias convencionais desses pacientes em dois momentos: quando do diagnóstico e após o término do tratamento. Foram considerados sinais compatíveis com atividade de tuber-culose: nódulos centrolobulares de distribuição segmentar, micronódulos confluentes, consolidações, cavidades de paredes espessas, nódulos, massas, espessamento de paredes brônquicas, aspecto de árvore em florescência e bronquiectasias cilíndricas.

**VARIÁVEIS ESTUDADAS:**

Comparou-se a presença de sinais compatíveis com atividade de tuberculose no início do tratamento e após o término do tratamento através do teste dos sinais (z).

**RESULTADOS:**

Todos os pacientes (20/20) apresentavam sinais compatíveis com atividade de tuberculose no início do tratamento. Após o término do tratamento, 13 pacientes (13/20) ainda apresentavam algum sinal sugestivo de atividade. Houve diminuição da extensão do acometimento pulmonar no pós-tratamento em relação ao início do tratamento (z = 10,10). Essa mudança foi estatisticamente significativa (p < 0,001).

**CONCLUSÃO:**

Os sinais sugestivos de atividade da tuberculose, na tomografia computadorizada do tórax, estão presentes na doença ativa e a extensão do parênquima acometido diminui significativamente após o término do tratamento.

## INTRODUCTION

Tuberculosis is the most common infectious disease afflicting the human species, affecting 8.4 million people throughout the world in 1999. It has been estimated that this number will rise to 10.2 million by 2005, with the majority of cases occurring in emerging countries.^1^ In Brazil, 90,000 new cases are reported each year, with an estimated 130,000 active at present.^2^

The diagnosis of pulmonary tuberculosis in Brazil is based upon two positive direct bacilloscopy findings in the sputum, or a positive culture for *Mycobacterium tuberculosis*. In the absence of these discoveries, suggestive radiological images or other complementary associated tests based upon clinical discoveries are indicative of active disease.^3^ Adequate knowledge of images consistent with tuberculosis activity is therefore an important resource for its diagnosis and treatment, particularly in those cases in which it is not possible to achieve bacteriological confirmation.

Chest radiography remains the primary imaging technique for the diagnosis and follow-up of pulmonary tuberculosis. However, computed tomography can help identify or confirm the presence of findings that may be used to suggest a tuberculosis diagnosis when the radiographic findings are inconsistent but tuberculosis is suspected clinically.

Computed tomography of the thorax is used in cases of clinical suspicion of pulmonary tuberculosis, particularly in those cases in which the initial thoracic radiography appears normal, as well as in differentiating this from other thoracic diseases and aids or fever of unknown origin.^2,4^ In a study of 42 patients with tuberculosis confirmed by bacteriological tests, Campos et al.^5^ concluded that high resolution computed tomography can be strongly suggestive of pulmonary disease activity. This is particularly helpful in patients with negative smear and/or indeterminate radiograms and allows proper treatment to be established, even before mycobacteria are identified via culturing.

In a study performed by Lee et al.,^6^ the diagnosis of tuberculosis through high resolution computed tomography was accurate in 88% of the patients (165/188), for ruling out or confirming the pulmonary disease. Other studies have confirmed that computed tomography is superior to thoracic radiography in the initial evaluation of tuberculosis.^7-9^

The objective of this study was to utilize conventional computed tomography for evaluating the structural alterations in the pulmonary parenchyma caused by tuberculosis, during the active phase of the disease and also after concluding the treatment.

## METHODS

Twenty patients with pulmonary tuberculosis and serologically negative for the HIV virus were prospectively studied. Tuberculosis was confirmed by positive culturing for *Mycobacterium tuberculosis* in sputum (19 patients) or via a lung fragment obtained through transbronchial biopsy (one patient).

All patients were informed of the procedures to be performed, and were subsequently submitted to conventional computed tomography at Hospital das Clínicas of the Universidade de São Paulo. Tomographic images were obtained using a conventional Phillips Tomoscan LX tomographic scanner (axial cuts of 10 mm thickness, in 10-mm increments from the apical area to the base of the lungs).

The study protocol was approved by the Ethics Committee for Research Project Assessment at Hospital das Clínicas of the Universidade de São Paulo.

The tomographic evaluation performed was based on two separate time periods: the first, from the diagnosis of tuberculosis until 30 days after the start of treatment with rifampicin, isoniazid and pyrazinamide; and the second, until 30 days after the completion of the proposed treatment (six months), when all the patients were considered to be cured, according to clinical criteria. The images were analyzed by three observers.

Suggestive signs of tuberculosis activity included: centrilobular nodules with segmental distribution, confluent micronodules, consolidations, thick-walled cavities, nodules, masses, thickening of the bronchial walls, tree-in-bud appearance and cylindrical bronchiectasis.^3,5,9-13^

For the analyses of the images obtained, the lungs were divided into three sections: upper, middle and lower. Furthermore, each section was divided into two parts: anterior and posterior. Analyses of 240 fields were performed (12 fields each patient) at the two study times.

The tomographic findings were classified into three grades in accordance with the observed extent of one or more of the signs of tuberculosis activity within each field analyzed:

Grade 0: absence of suggestive signs of tuberculosis activity.

Grade 1: presence of suggestive signs of tuberculosis activity in up to 50% of the analyzed field.

Grade 2: presence of suggestive signs of tuberculosis activity in more than 50% of the analyzed field.

The sensitivity and specificity of this method were calculated. The presence of signs of tuberculosis activity, as shown by computed tomography, was compared between the start and completion of the treatment using the signs test (z). The significance level of 5% was adopted.

## RESULTS

The patients’ mean age was 34.3 years (range: 16 to 71 years old) with a standard deviation of 13.8 years. Of the 20 individuals studied, eleven were male (55%) and nine were female (45%). All the patients presented symptoms clinically consistent with tuberculosis.

From computed tomography, all patients (20/20) presented signs consistent with tuberculosis activity at the start of the treatment. After completion of the treatment, seven patients did not present any of the previously noted signs (7/20), while 13 patients (13/20) still presented some suggestive signs of activity. The sensitivity of the computed tomography was 100%, while the specificity was 35%.

**Figure 1:**
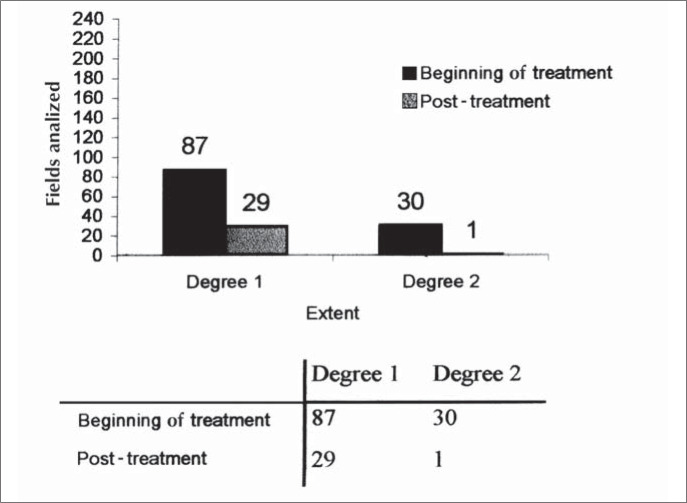
Histogram of extent of parenchyma attack at the beginning and post-treatment.

Compromised parenchyma (disease extent of grade 1 or 2) was observed in 117 fields of the 240 analyzed at the start of treatment, and in only 30 fields after the end of treatment (Figure 1).

**Figure 2:**
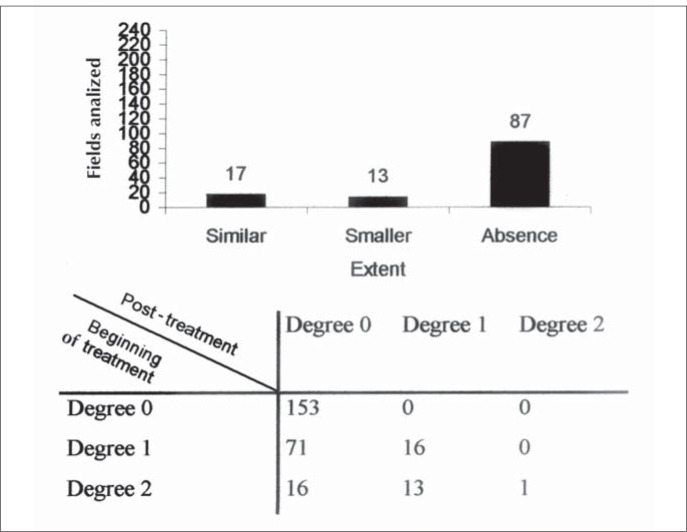
Histogram of extent of CT Scan findings at post-treatment in those fields with parenchyma attack at the beginning of treatment.

Upon the completion of treatment, it was observed that in the fields still presenting signs of tuberculosis activity, as seen via computed tomography, the extent of the lesions was similar in 17 of the analyzed fields (grades 2/2 or 1/1). In 13 fields, the extent was less than was observed at the start of the treatment (grade 2/1). In 87 fields, there was an absence of suggestive signs of tuberculosis activity, when such signs had been present at the start of the treatment (grades 2/0 or 1/0) (Figure 2).

There was a decrease in the extent of suggestive signs of tuberculosis activity observed via computed tomography after the end of the treatment, in comparison with what had been observed at the start of the treatment (z = 10.10). This change was statistically significant (p < 0.001).

The tomographic suggestive signs of tuberculosis found in the 20 patients are described in Table 1.

**Table 1:** Tomographic findings from 20 patients with pulmonary tuberculosis with suggestive signs at the start of treatment and post-treatment

Computed tomography findings	Start of treatment		Post-treatment		Post-treatment/start of treatment	
	N	%	N	%	N	%
Thick-walled cavities	16	80.0	1	5.0	1/16	6.2
Centrilobular nodules	19	95.0	5	25.0	5/19	26.3
Confluent micronodules	16	80.0	0	-	0/16	-
Nodules	14	70.0	7	35.0	7/14	50.0
Consolidations	9	45.0	3	15.0	3/9	33.3
Masses	12	60.0	9	45.0	9/12	75.0
Thickening of bronchial walls	13	65.0	4	20.0	4/13	30.7
Tree-in-bud appearance	12	60.0	1	5.0	1/12	8.3
Cylindrical bronchiectasis	4	20.0	0	-	0/4	-
Thin-walled cavities	0	-	5	25.0	5/0	-
Traction bronchiectasis	0	-	7	35.0	7/0	-
Bands	2	10.0	14	70.0	14/2	-

## DISCUSSION

In the initial phase of *Mycobacterium tuberculosis* infection, the inhaled bacilli reach the alveoli, where a process of nonspecific inflammation takes place, mediated by alveolar neutrophils and macrophages. The release of oxidizing substances and elastin creates a core of pointed alveolar exudate, characterized by necrosis of the alveoli, fibrin exudation, degenerate neutrophils and a great number of viable bacilli.^12-14^ This, the host's first line of defense, determines the formation of exudative nodules. The filling of the alveoli by a material that is denser than air is defined in radiology as consolidation.^15,16^ As such, this first stage of the infectious process can be radiologically identified as small nodules and consolidations in the pulmonary parenchyma. During the present study, an appearance of segmental or lobular consolidation was observed via computed tomography in 45% of the cases, as presented in Table 1.

If the exudative response is not sufficient to contain the progress of the bacilli, macrophages are activated and cause phagocytosis of the bacilli. This introduces antigens of the microorganism to the lymphoid tissue associated with the bronchus. The activated macrophages in the alveoli crowd around the bacilli and become epithelium cells, which themselves group together to form the gigantic multinuclear cells that form the granuloma of the tuberculosis.^14^

Groupings of formed granulomas are referred to as Ghon nodules. Groups of Ghon nodules constitute the primary tuberculosis complexes, which are known as lymphangitis and lymphadenitis. These may develop into the cure or the disease itself, depending upon the number and virulence of the bacilli, as well as the degree of host hypersensitivity and resistance.^17^

**Figure 3:**
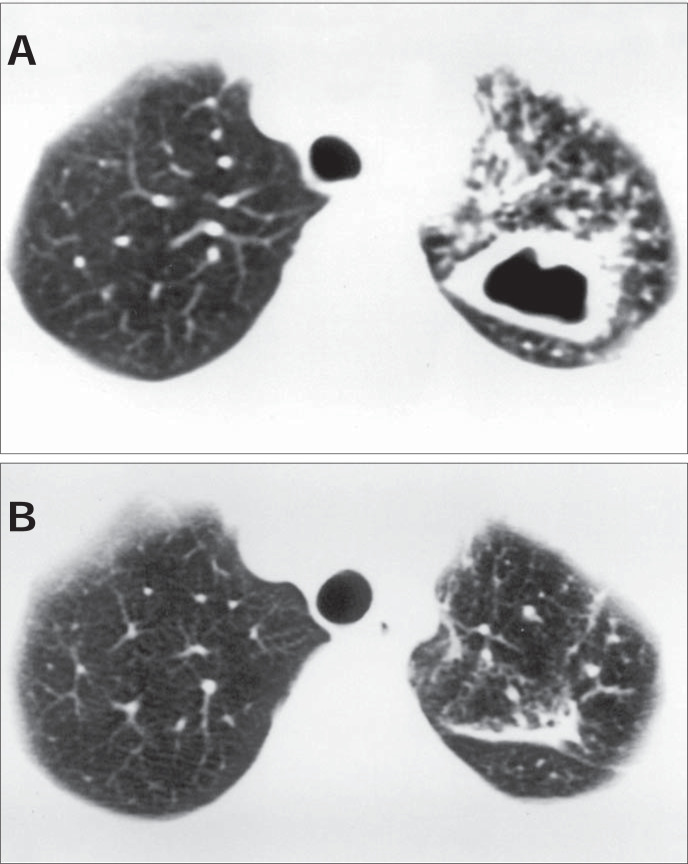
(A) Computed tomography at the start of treatment: thick-walled cavity in the left lung. (B) Computed tomography post-treatment, for the same patient: there is a band in the place where a cavity had been observed at the start of treatment.

The disease begins from the initial parenchymatous or ganglionic lesion. After necrosis of the lesion center, liquefaction follows (the elimination of liquid material by bronchial drainage and subsequent formation of cavities). These thick-walled cavities appear during the active phase of the tuberculosis. During the present study, thick-walled cavities were observed upon diagnosis in 80% of the patients (Figure 3).

**Figure 4:**
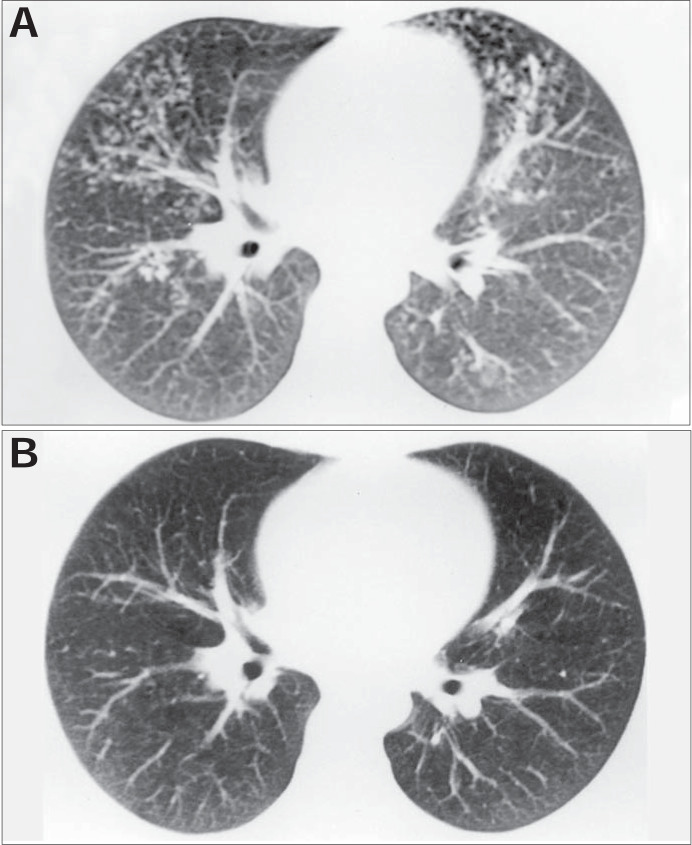
(A) Computed tomography at the start of treatment: centrolobular nodules with segmental distribution in the anterior parts of both lungs. (B) Computed tomography post-treatment, for the same patient: absence of the alterations observed at the start of treatment.

Centrilobular nodules with segmental distribution, representative of the bronchogenic spread of tuberculosis, are most frequently discovered through tomographic scanning in the active phase of the disease, and are present in up to 82% of the cases.^5,6^ In the present study, they were seen on 95% of the tomographic scans analyzed in the start of treatment (Figure 4).

These nodules tend to converge or form larger nodules and masses in 62% to 71% of the cases.^6^ In our sample, 80% and 70% of the patients presented confluent micronodules and larger nodules, respectively. Massive appearance was observed in 60% of the patients.

Thickening of the bronchial walls and cylindrical bronchiectasis are described in the literature in 62% and 23% of the patients with active pulmonary tuberculosis, respectively. Tree-in-bud appearance is present in up to 57% of the cases.^2,5,6^ During the present study, 65% of the patients showed thickened bronchial walls. Bronchiectasis was present in 20% and the tree-in-bud appearance was observed in 60% of the cases.

A variety of sequelae and complications can occur in pulmonary tuberculosis in treated or untreated patients. These can be categorized as parenchymal or airway lesions, which include thin-walled cavities, bands and bronchiectasis.^18^ The cavities are formed by scarring. The residual characteristics of such cavities include grooving, calcification and retraction of the attacked parenchyma. The cavities may also remain after curing, with their walls thinned, which represents the succession or inactivation of that specific process.^19,20^ Some authors suggest that, in radiology, these findings should be described as “stable” rather than “inactive”, because of the possibility of future recrudescence of latent bacilli.^21^

Such residual cavities and bronchiectasis may be colonized by *Aspergillus* species, nontuberculous mycobacteria or other microorganisms. Hemoptysis may be the clinically most important consequence of these sequelae. After the completion of treatment, thin-walled cavities were present in 25%, traction bronchiectasis in 35% and bands in 70% of the patients appraised in our study. These post-treatment findings, in relation to the initial conditions (Table 1), provide important evidence for the follow-up of patients with pulmonary tuberculosis. Therefore, recognition of radiological manifestations of the pulmonary sequelae is important for facilitating the understanding of complications due to the disease.^18,22^

## CONCLUSIONS

We have concluded that signs suggestive of tuberculosis activity are present in the active disease, and are seen via computed tomography. The extent of the parenchyma attack decreases significantly upon completion of the treatment. Such findings may be useful in the diagnosis of pulmonary tuberculosis, particularly when it is not possible to achieve bacteriological confirmation.
